# Supplementary material for: Patterns of Intron Gain and Loss in Fungi
Source: PLoS Biol. 2004 Nov 30;2(12):e422. doi: 10.1371/journal.pbio.0020422 (PMC532390; doi:10.1371/journal.pbio.0020422)
Supplement: Table S1 — Also available at http://genes.mit.edu/NielsenEtAl/. (4.3 MB ZIP). [file pbio.0020422.st001.zip › NielsenEtAl/html/454.html]

AN0316.1.NCU09468.1.MG06650.1.FG00639.1


```
 CLUSTAL W (1.82) Multiple Sequence Alignments - Introns Inserted


Sequence 1: NCU09468.1	449 aa
Sequence 2: FG00639.1	449 aa
Sequence 3: AN0316.1	449 aa
Sequence 4: MG06650.1	461 aa
Alignment Length: 461 aa
Number Identitical Residues: 372 aa
Alignment Score (without introns) 15551


MG06650.1 	MREIISINGKRV~SKQNSQDSSPHQPRTRHSSNT0LYCLEHGIK0PDGFLTDERKAADPD
NCU09468.1	MREIISLN---V1GQAGCQIAN--------SCWE0LYCLEHGIQ0PDGYLTEERKAADPD
FG00639.1 	MREVISIN---V1GQAGCQIAN--------SCWE0LYCLEHGIQ0PDGYLTEERKAQDPD
AN0316.1  	MREVISLN---V1GQAGCQIAN--------SCWE0LYCLEHGIQ0PDGYLTEERKKEDPD
          	***:**:*   * .: ..* :.        *.   ********: ***:**:***  ***

MG06650.1 	HGFSTFFSETG1QGKYVPRTIYCDLEPNVVDEVRTGTYRSLFHPEQMITGKEDASNNYAR
NCU09468.1	HGFSTFFSETG1NGKYVPRTIYADLEPNVIDEVRTGAYRGLFHPEHMISGKEDASNNYAR
FG00639.1 	QGFSTFFSETG1NGKHVPRAIYCDLEPNVVDEVRTGAYRNLFHPEMMITGKEDASNNYAR
AN0316.1  	HGFSTFFSETG1QGKYVPRTIYADLEPNVVDEVRTGTYRSLFHPENLITGKEDASNNYAR
          	:********** :**:***:**.******:******:**.***** :*:***********

MG06650.1 	GHYTVGKELIDQVLDKVRRVADSCSGLQGFLVFHSFGGGTGSGFGSLLMERLSVDYGKKS
NCU09468.1	GHYTVGKELIDQVLDKVRRVADNCSGLQGFLVFHSFGGGTGSGFGALLMERLSVDYGKKS
FG00639.1 	GHYTVGKELIDGVLDKIRRVADNCAGLQGFLVFHSFGGGTGSGFGALLMERLSVDYGKKS
AN0316.1  	GHYTVGKEMIDQVLDKVRRMADSCSGLQGFLVFHSFGGGTGSGFGALLMERLSVDYGKKS
          	********:** ****:**:**.*:********************:**************

MG06650.1 	KLEFCVYPAPQTATSVVEPYNSILTTHTTLEHSDCSFMVDNEAIYDICRRSLGLERPNYE
NCU09468.1	KLEFCVYPAPQTATSVVEPYNSILTTHTTLEHADCSFMVDNEAIYDICRRNLGLERPNYE
FG00639.1 	KLEFCVYPAPQTATSVVEPYNSILTTHTTLEHSDCSFMVDNEAIYDICRRNLGLERPNYE
AN0316.1  	KLEFCVYPAPQNATSVVEPYNSILTTHTTLEHSDCSFMVDNEAIYDICRRNLGIERPSYE
          	***********.********************:*****************.**:***.**

MG06650.1 	NLNRLIAQV1VSSITASLRFDGSLNVDLNEFQTNLVPYPRIHFPLVAYSPIVSAARAAHE
NCU09468.1	NLNRLIAQV1VSSITASLRFDGSLNVDLNEFQTNLVPYPRIHFPLVAYAPVISAAKAAHE
FG00639.1 	NLNRLIAQV1VSSITASLRFDGSLNVDLNEFQTNLVPYPRIHFPLVAYSPVISAAKAAHE
AN0316.1  	NLNRLIAQV~VSSITASLRFDGSLNVDLNEFQTNLVPYPRIHFPLVAYSPVISADKASHE
          	********* **************************************:*::** :*:**

MG06650.1 	ANSVQEITMSCFEPNNQMVKCDPRNGKYMATCLLFRGDVVPKDAHAAVAMLKTKRSIQFV
NCU09468.1	ANSVQEMTMSCFEPNNQMVKCDPRHGKYMATCLLYRGDVVPNDAHAAVATLKTKRTIQFV
FG00639.1 	ANSVQEMTMSCFEPNNQMVKCDPRHGKYMATCLLYRGDVVPNDAHAAVATLKTKRTIQFV
AN0316.1  	ANSVQDITMSCFEPNNQMVKCDPRNGKYMATCLLYRGDVVPKETHAAVATLKTKRTIQFV
          	*****::*****************:*********:******:::***** *****:****

MG06650.1 	DWCPTGFKLGICFEPPQQVPNGDLAKVNRAV2CVLSNTTAIADAWSALSNKFDLMYSKRA
NCU09468.1	DWCPTGFKLGICYQPPHQVPNGDLAKVNRAV2CMLSNTTAIAEAWSALSSKFDLMYSKRA
FG00639.1 	DWCPTGFKLGICYQAPENVPNGDLAKVSRAV2CMLSNTTAIAEAWSSLSLKFDLMHSKRA
AN0316.1  	DWCPTGFKIGICYQPPQQVPNGDLAKVNRAV2CMLSNTTAISEAWSALDHKFDLMYSKRA
          	********:***::.*.:*********.*** *:*******::***:*. *****:****

MG06650.1 	FVHWYVGEGMEEGEFSEAREDLAALERDYEEVATDSLGDEEGIEAEY
NCU09468.1	FVHWYVGEGMEEGEFSEAREDLAALERDYEEVAADSMEGED-VEAEY
FG00639.1 	FVHWYVGEGMEEGEFSEAREDLAALERDYEEVATDSMGDEE-LEAEY
AN0316.1  	FVHWYVGEGMEEGEFSEAREDLAALERDYEEVASDSLEEEG-EEVEY
          	*********************************:**:  *   *.**
```
